# The association between periodontal diseases and helicobacter pylori: an updated meta-analysis of observational studies

**DOI:** 10.1186/s12903-023-03232-3

**Published:** 2023-07-26

**Authors:** Yousef Moradi, Lobat Majidi, Sorour Khateri, Nima Azh, Reza Ghanei Gheshlagh, Nadia Saniee, Mostafa Zarei, Farhad Moradpour

**Affiliations:** 1 Social Determinants of Health Research Center, Research Institute for Health Development, Kurdistan University of Medical Sciences, Sanandaj, Iran; 2 Department of Physical Medicine and Rehabilitation, School of Medicine, Hamedan University of Medical Sciences, Hamedan, Iran; 3 School of Medicine, Iran University of Medical Sciences, Tehran, Iran; 4 Spiritual Health Research Center, Research Institute for Health Development, Kurdistan University of Medical Sciences, Sanandaj, Iran; 5 Department of Public Health, Asadabad School of Medical Sciences, Asadabad, Iran

**Keywords:** Helicobacter pylori, Periodontal disease, Systematic review, Meta-analysis

## Abstract

**Introduction:**

Various studies have examined the association between periodontitis and helicobacter pylori and reported conflicting results. The aimed of this systematic review and meta-analysis estimating the association between these two variables.

**Methods:**

Electronic databases including PubMed (Medline), Scopus, Web of Sciences and Medline (Elsevier) were searched using the relevant keywords. All observational studies comparing the association between periodontitis and helicobacter pylori were considered. The Newcastle - Ottawa Quality Assessment Scale (NOS) checklist was used for assessing quality of included studies. All statistical analyses were completed using STATA (Version 16).

**Results:**

Twenty-three studies with 8,638 patients (15 case-control with 2,366 patients and 8 cross-sectional with 6,272 patients) were included in this meta-analysis. After combining the selected studies, the odds of presence the Helicobacter pylori infection in patients with the periodontal disease was 2.47 (OR: 2.47; 95% CI: 2.01, 3.03; I^2^: 50.87%; *P*: 0.001). Also, the odds after combining case-control studies was 2.77 (OR: 2.77; % 95 CI: 2.11, 3.66; I^2^: 37.16%; *P*: 0.049) and after combining cross-sectional analytical ones, it was equal to 2.07 (OR: 2.07; 95% CI: 1.62, 2.65; I^2^: 43.25%; *P*: 0.050).

**Conclusion:**

Based on the results of this meta-analysis, the association between Helicobacter pylori infection and the periodontal disease is evident.

**Supplementary Information:**

The online version contains supplementary material available at 10.1186/s12903-023-03232-3.

## Introduction

Helicobacter pylori (*H. Pylori*) is one of the most common bacterial infections in humans, associated with chronic gastritis, gastric ulcers and gastric cancers [1]. *H. Pylori* is estimated to affect about half of the world’s population and is considered a major cause of chronic gastritis and peptic ulcers. Eradication of *H. Pylori* infection improves wound healing, prevents recurrence, and reduces the incidence of *H. Pylori*-related gastric diseases [2–4]. According to previous studies, this bacterium is a risk factor for some oral diseases, such as periodontal diseases (PDs), canker sores, squamous cell carcinoma, tongue irritation and bad breath [1, 5]. The oral cavity can be an important reservoir for this infection and oral secretions may be an important means of transmitting this microorganism [6]. *H. Pylori* is the cause of many oral diseases, including chronic periodontitis (CP). Gingival inflammation, bleeding, periodontal pocket formation, alveolar bone resorption, alveolar bone height reduction, and tooth loss are common symptoms of chronic periodontitis [7]. At present, prophylactic treatment focuses on the removal of dental plaque [7]. Findings of meta-analysis by Wei et al. showed the incidence of *H. Pylori* in patients with the PDs was 3.42 times higher than the control group [7]. In one study, the effect of PDs treatment on *H. Pylori* was investigated and the results showed a significant reduction in *H. Pylori* among patients who received PDs treatment. In PDs treatment, the microbes colonized on the tooth surface are removed by the dentist and people are recommended to use dental floss and mouthwash to control plaque [8]. According to the latest research, the incidence of *H. Pylori* in people with the PDs is significantly higher than normal people, and in many cases, *H. pylori* is found in pockets created in the PDs. In other words, dental plaque and pockets formed in the mouth can cause *H. pylori* bacteria to accumulate in them and cause recurrence of the disease over time. The presence of this bacterium in the mouth can also increase the depth of oral pockets and the degree of periodontal damage. Since *H. Pylori* infection is completely dependent on the oral general condition and health and seriously uses the created empty spaces, maintaining proper oral hygiene and regular removal of plaque can be said to have an important effect on the control of *H. pylori* infection in addition to preventing the PDs [9–13].

Experts have also concluded periodontal treatment as an adjunct can have significant short-term and long-term effects in the treatment and eradication of *H. pylori*. In fact, periodontal treatment can be an additional treatment for patients with *H. pylori* and can help eradicate this bacterium [14, 15].

Various studies have examined the association between periodontitis and *H. pylori* and reported conflicting results. In some studies, there was no association between these two variables while in some other ones, an association between these variables was reported [16–20]. Even though several meta-analysis studies published so far, the association between periodontitis and *H. pylori* in general population not considered [14, 15, 20]. Also, determining the exact association between these two factors can have beneficial effects on updating treatment and care guidelines as well as can make appropriate and effective changes in the first line of treatment in both diseases. Accordingly, the present systematic review and meta-analysis was performed with the aim of estimating the association between these two variables.

## Methods

This systematic review and meta-analysis was written and reported based on the checklist of the Preferred Reporting Items for Systematic Reviews and Meta-Analyzes (PRISMA) [21]. The structure of this meta-analysis included search strategy, article screening, final article selection, data extraction, quality assessment, and data analysis.

### Search strategy and article screening

To perform this meta-analysis, the search keywords were first selected, which included “helicobacter pylori” and “periodontal diseases”. Synonyms for the keywords were then selected from the mesh, Emtree, and Thesaurus databases. These keywords were included “helicobacter nemestrinae”, “campylobacter pylori”, “campylobacter pylori subsp. Pylori”, “parodontosis”, “parodontoses”, and “pyorrhea alveolaris”. In the next step, search syntaxes were compiled (Supplement file Table S1) and adapted for each of the international databases including PubMed (Medline), Scopus, Web of Sciences and Medline (Elsevier). In addition to these databases, manual search was performed by hand-searching and perusing reference lists (reviewing the references of related and selected articles in order to obtain other related studies). The target time range for the search was from January 1990 to May 2022. After completing the search, all articles from the desired databases were entered into the Endnote software (version 8) and then duplicates were removed. The remaining studies were screened based on their titles, abstracts and full texts. Screening was performed according to the inclusion criteria including (Table 1). In this meta-analysis, we followed the PECOT structure and selected primary studies that had an all-population study population, considered people with PDs as the exposure group, people without PDs as the comparison group, and the occurrence of *H. pylori* as the desired outcome. The reason for not choosing cohort studies was the small number of these studies related to the subject of this meta-analysis. Other studies such as systematic reviews, clinical trials, laboratory studies, animal studies, letters to the editor, or short communications were excluded. Articles other than English language and inaccessible ones were also removed from the study. Article screening was done completely and independently by the two authors (MZ and SKH) and disputes were resolved by discussion and referral to the third party (YM) if necessary.

**Table 1:** Criteria for Studies Included in this Meta-Analysis

Population (P)	Exposure (E)	Comparison (C)	Outcomes (O)	Type of Study (T)
The target population in this meta-analysis was all people (without any restriction).	The desired exposure in the present meta-analysis was periodontal diseases.	The comparison group was people without periodontal diseases.	The desired outcome was the occurrence of H. Pylori infection.	All analytical cross-sectional and case-control studies were considered.

### Data extraction

In order to extract data, first the opinions of all authors about the items and variables to be extracted from selected articles were collected. Then, a checklist was designed including the author’s name, year of publication, country of the study, type of the study, age, study population, sample size, method of diagnosis of *H. pylori* infection and sampling location. All steps of data extraction were independently performed by the two authors and in case of any differences, they were resolved with the third person’s opinion.

### Quality assessment

Qualitative evaluation of the studies was conducted by two of the authors (YM and FM) based on the Newcastle - Ottawa Quality Assessment Scale (NOS) checklist designed to evaluate the quality of observational studies [22]. It was developed to assess the quality of non-randomized studies (case-control, cohort, and cross-sectional studies) with its design, content and ease of use directed to the task of incorporating the quality assessments in the interpretation of meta-analytic results. This tool examines each research with 8 items in three groups, including how to select study samples, how to compare and analyze study groups, and how to measure and analyze the desired outcome. Each of these items is given a score of one if it is observed in the studies, and the maximum score for each study is 9 points. In case of discrepancies in the score assigned to the published articles, the discussion method and the third researcher were applied to reach an agreement. Results of quality assessment mentioned in supplement file (Supplement file Tables S2 and S3).

### Data analysis

To calculate the association, cumulative odds ratio (OR) with the 95% confidence interval and the meta set command were used considering logarithm and logarithm standard deviation of the OR. In this meta-analysis, the Der Simonian–Laird random-effects model (REM) was used to estimate pooled OR with a 95% confidence interval (95% CI). Heterogeneity was assessed between studies using the I^2^ and Q Cochrane tests. According to Cochrane’s reported criteria, 0 to 25% indicate no heterogeneity, 25 to 50% indicate low heterogeneity, 50 to 75% indicate high but acceptable heterogeneity and 75 to 100% indicate high and unacceptable heterogeneity [23, 24]. Egger test and funnel plot were used to evaluate the publication bias. For detecting publication bias, if the P-value of Egger test lower than 0.05, authors could conclude the publication bias was occurred. Statistical analysis was performed using STATA 16.0 and *P*-value < 0.05 was considered. the Grading of Recommendations Assessment, Development, and Evaluation (GRADE) approach was employed to evaluate the overall quality of evidence for each outcome in the summary of findings table. The GRADEpro GDT online software was utilized for the GRADE approach and to create the summary of findings Tables [25].

## Results

**Fig. 1:**
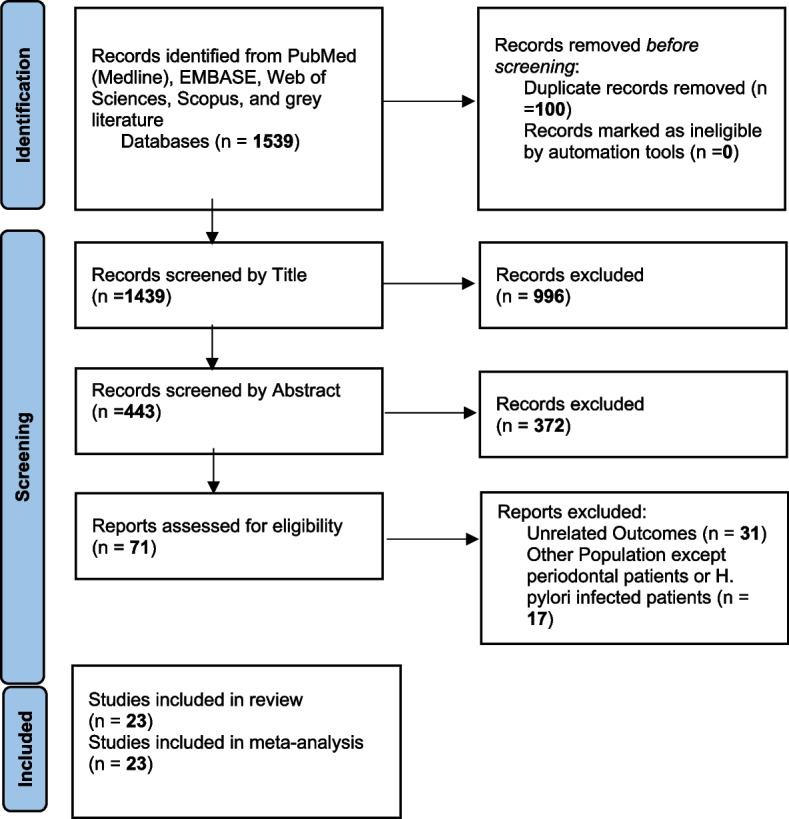
Identification of studies via databases and registers

After removing duplicate articles, 1,439 studies were entered into the screening stage based on the title. Then, 996 articles were removed and 443 ones were screened based on the abstract. In the next stage, 71 articles remained and were screened based on the full text. Finally, 23 studies were entered into the present meta-analysis (Table 2; Fig. 1).

**Table 2:** The characteristics of included studies (Case-Control and Cross- Sectional Studies)

Authors	Years	Design	Study	Sample Size	Population	Age	Country	A	B	C	D	Methods	Sample	NOS
**Nisha KJ, et al. **[26]	2016	Cross Sectional	Community	499	General Population	43.67	India	180	113	90	117	Rapid Urease Test	Oral Sample	7
**Nisha KJ, et al. **[26]	2016	Cross Sectional	Community	500	General Population	43.67	India	209	84	136	71	Serology	Oral Sample	6
**Dye BA, et al. **[19]	2002	Cross Sectional	Community	4504	General Population		USA	202	951	291	3030	ELISA	Oral Sample	8
**LX Gong, et al. **[3]	2011	Case Control	Community	562	Chronic Periodontal		China	438	58	41	25	Rapid Urease Test	Oral Sample	8
**Zheng P, et al. **[28]	2015	Case Control	Community	140	Elderly Population	63.8	China	40	30	24	46	Polymerase Chain Reaction (PCR)	Oral Sample	7
**MY Wang, et al. **[5]	2015	Case Control	Community	200	Chronic Periodontal		China	103	17	59	21	Rapid Urease Test	Oral Sample	7
**Jing Li, et al. **[30]	2015	Case Control	Community	176	Chronic Periodontal		China	69	16	52	37	Rapid Urease Test	Oral Sample	8
**Umeda M, et al. **[31]	2003	Cross Sectional	Hospital	36	Chronic Periodontal	54.5	Japan	7	1	10	10	Polymerase Chain Reaction (PCR)	Oral Sample	7
**Anand PS, et al. **[1]	2006	Case Control	Hospital	134	Gastric Patients	40	India	30	20	35	49	Rapid Urease Test	Oral Sample	7
**Souto R, et al. **[32]	2008	Cross Sectional	Hospital	225	Chronic Periodontal	39.3	Brazil	40	4	129	52	Polymerase Chain Reaction (PCR)	Oral Sample	8
**Al Asqah M, et al. [**33]	2009	Case Control	Hospital	101	Gastric Patients	40.77	Saudi Arabia	49	17	13	22	Rapid Urease Test	Oral Sample	8
**Al Asqah M, et al. **[33]	2009	Case Control	Hospital	101	Gastric Patients	40.77	Saudi Arabia	37	17	13	22	Rapid Urease Test	Stomach	8
**Medina ML, et al. **[34]	2010	Case Control	Hospital	98	Gastric Patients	44	Argentina	15	3	28	52	Polymerase Chain Reaction (PCR)	Oral Sample	8
**Silva DG, et al. **[18]	2010	Cross Sectional	Hospital	115	Gastric Patients	50	Brazil	36	22	31	26	Polymerase Chain Reaction (PCR)	Oral Sample	6
**Salehi MR, et al. **[16]	2013	Case Control	Hospital	100	Chronic Periodontal	35.53	Iran	9	12	41	38	Polymerase Chain Reaction (PCR)	Oral Sample	6
**Sujatha S, et al. **[35]	2015	Cross Sectional	Hospital	40	Gastric Patients	45.15	India	26	2	6	6	Rapid Urease Test	Oral Sample	7
**Yang J, et al. **[36]	2016	Case Control	Hospital	212	Chronic Periodontal	56.5	China	78	58	25	51	Polymerase Chain Reaction (PCR)	Oral Sample	7
**Tahbaz SV, et al. **[37]	2017	Case Control	Hospital	100	Chronic Periodontal	54.5	Iran	4	1	46	49	Polymerase Chain Reaction (PCR)	Oral Sample	7
**Riggio MP, et al. **[38]	1999	Cross Sectional	Hospital	73	Chronic Periodontal	45.1	UK	11	18	13	31	Polymerase Chain Reaction (PCR)	Oral Sample	7
**Li Wang, et al. **[18]	2001	Case Control	Hospital	106	Chronic Periodontal		China	21	41	4	40	Polymerase Chain Reaction (PCR)	Oral Sample	7
**Al-Refai AN, et al. **[40]	2002	Case Control	Hospital	135	Chronic Periodontal	37.2	Saudi Arabia	67	8	52	8	Rapid Urease Test	Oral Sample	8
**YH Jiang, et al. **[20]	2002	Case Control	Hospital	60	Chronic Periodontal		China	29	11	7	13	Polymerase Chain Reaction (PCR)	Oral Sample	6
**Jing Gao, et al. **[42]	2011	Case Control	Hospital	76	Chronic Periodontal		China	24	13	15	24	Polymerase Chain Reaction (PCR)	Oral Sample	7
**A Tsimpiris, et al. **[43]	2021	Case Control	Hospital	65	Chronic Periodontal	55.5	Greece	6	27	7	25	Polymerase Chain Reaction (PCR)	Oral Sample	7
**Almashhadany DA, et al. **[44]	2022	Cross Sectional	Hospital	280	Chronic Periodontal	51.7	Iraq	64	49	53	114	Rapid Urease Test	Oral Sample	7

A: Exposed cases (the number of patients with H. Pylori in all the PDs patients), B: Exposed non-cases (the number of patients with H. Pylori in the people without PDs), C: Unexposed cases (the number of people without H. Pylori in all the PDs patients), D: Unexposed non-cases (the number of the people without H. Pylori in the people without PDs), NOS: the Newcastle - Ottawa Quality Assessment Scale,

Out of the selected articles, there were 8 cross-sectional and 15 case-control studies to determine the association between *H. pylori* infection and the PDs, of which 17 studies were conducted in Asia, 4 in the United States and 2 in Europe (Table 2).

**Fig. 2:**
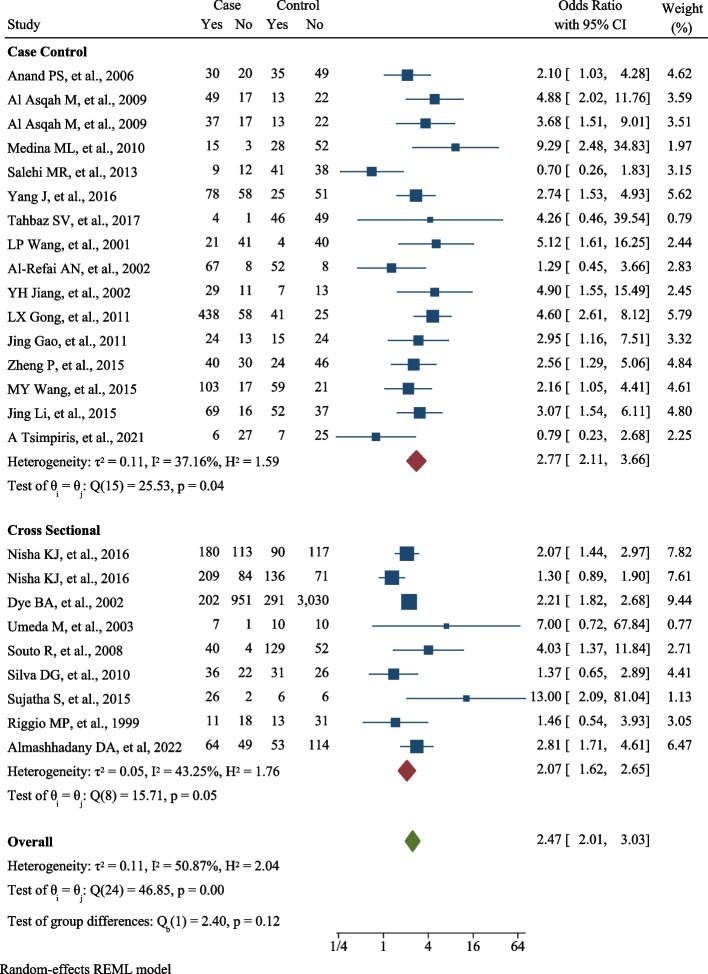
The pooled Odds Ratio for association between H. pylori infection and Periodontal diseases occurrence by type of studies

**Fig. 3:**
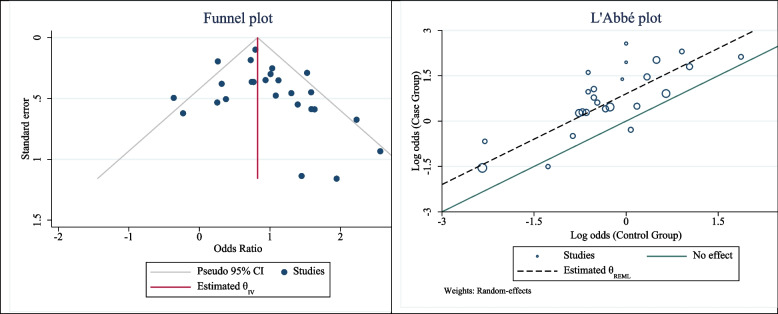
Funnel plot and heterogeneity assessment of association between H. pylori infection and Periodontal diseases occurrence

After combining the selected studies, the odds ratio of *H. pylori* infection in patients with the PDs was 2.47 (OR: 2.47; 95% CI: 2.01, 3.03; I^2^: 50.87%; P value: 0.001). The odds ratio after combining case-control studies was 2.77 (OR: 2.77; % 95 CI: 2.11, 3.66; I^2^: 37.16%; *P*: 0.049) and after combining cross-sectional analytical ones, it was equal to 2.07 (OR: 2.07; 95% CI: 1.62, 2.65; I^2^: 43.25%; *P*: 0.050) (Fig. 2). The publication bias was evaluated using the eggers and funnel plot tests and the test results showed it did not occur (B: 0.201; SE: 0.09; *P*: 0.330). The results of funnel plot have been shown in Fig. 3. Results of the L’Abbé plot indicated that majority of selected studies are homogeneous, because they are near to the dashed line (except for two studies). Also, these results are in the line of I2 and Q Cochrane test results in main funnel plot (Fig. 3).

The results of subgroup analyze have been shown in Table 3. The results showed the odd ratio of *H. pylori* infection in patients with the PDs after combining hospital-based studies was equal to 2.64 (OR: 2.64; % 95 CI: 1.94, 3.59; I^2^: 43.98%; *P*: 0.050) and after combining population-base studies, it was equal to 2.29 (OR: 2.29; 95% CI: 1.71, 3.07; I^2^: 66.41%; *P*: 0.035). Also, the odd ratio of *H. pylori* infection in PDs patients who did not have any chronic disease, had the CPD and had gastrointestinal diseases such as gastric were equal to 1.94 (OR: 1.94; 95% CI: 1.49, 2.54; I^2^: 54.65%; *P*: 0.091), 2.57 (OR: 2.57; 95% CI: 1.93, 3.43; I2: 36.07%; P value: 0.070) and 3.94 (OR: 3.94; 95% CI: 1.89, 6.44; I^2^: 56.76%; *P*: 0.050), respectively. According to the method of diagnosing *H. pylori* infection, the odd ratio of *H. pylori* infection in PDs diagnosed with the Rapid Urease Test was higher than the odd ratio in other patients whose infection was diagnosed by other diagnostic methods (OR: 2.80; % 95 CI: 2.18, 3.60; I^2^: 22.85%; *P*: 0.140). American PDs patients were more likely to have *H. pylori* infection than Asian and European ones (Table 3).

**Table 3:** Subgroup analysis of the association between H. pylori infection and Periodontal diseases occurrence by type of study based, population, methods of sampling and detect

Variables	Subgroups	Number of study	Pooled Odds Ratio (95% CI)	Between Studies			Between Subgroup	
				I square (%)	P heterogeneity	Q	Q	*P* value
Type of study based	Hospital Based	18	2.64 (1.94–3.59)	43.98	0.05	30.29	0.42	0.59
	Community Based	7	2.29 (1.71–3.07)	66.41	0.03	14.25		
Population	General Population	4	1.94 (1.49–2.54)	54.65	0.09	6.43	3.87	0.43
	Chronic Periodontal	15	2.57 (1.93–3.43)	36.07	0.07	22.34		
	Gastric Patients	6	3.49 (1.89–6.44)	56.76	0.05	11.84		
H. pylori detection	Polymerase Chain Reaction (PCR)	13	2.47 (1.66–3.67)	48.01	0.04	22.14	2.65	0.27
	Rapid Urease Test	10	2.80 (2.18–3.60)	22.85	0.14	13.43		
	Other (Serology or ELIZA)	2	1.74 (1.04–2.92)	83.09	0.02	5.92		
Continents	Asia	19	2.60 (2.05–3.31)	47.43	0.01	37.22	4.11	0.13
	America	4	2.72 (1.45–5.25)	68.14	0.06	7.28		
	Europe	2	1.14 (0.53–1.47)	0.00	0.47	0.53		
Periodontal Disease detection	CPITN	1	1.29 (0.45–3.66)	0.00	-	0.00	28.55	0.00
	Index of Greene and Vermillion	1	2.10 (1.03–4.28)	0.00	-	0.00		
	Not reported	6	3.59 (2.51–5.13)	0.00	0.62	3.54		
	Oral examination	3	4.90 (2.81–8.52)	49.35	0.14	3.95		
	Probing test and clinical attachment loss	8	1.43 (1.13–1.82)	76.78	0.00	30.14		
	Probing test, Plaque index, Bleeding index	2	2.57 (1.36–4.85)	0.00	0.96	0.00		
Clinical attachment loss	Not Reported	13	3.17 (2.46–4.09)	42.70	0.09	14.98	26.75	0.00
	Attachment loss (mm)	2	3.04 (1.81–5.08)	0.00	0.57	0.32		
	> 4 mm	2	1.24 (0.86–1.79)	0.00	0.45	0.44		
	> 3 mm	4	1.27 (0.87–1.85)	24.76	0.29	9.17		
Pocket status	Not Reported	10	3.19 (2.43–4.18)	46.98	0.04	9.07	21.41	0.00
	Pocket depth (mm)	2	3.04 (1.81–5.08)	0.00	0.32	0.75		
	> 5	2	1.54 (0.79–3.03)	0.00	0.48	0.50		
	> 3	3	1.29 (0.81–2.05)	79.88	0.00	14.99		
	> 4	4	1.44 (1.05–1.98)	12.73	3.44	0.33		

Subgroup analysis based on various methods of periodontal disease (PD) detection revealed that the odds ratio of Helicobacter pylori (H. pylori) infection in PD cases diagnosed using the probing test and clinical attachment loss was higher in a greater number of studies compared to other methods. However, the odds ratio was lower than others (OR: 1.43; 95% CI: 1.13, 1.82; I^2^: 76.78%; *P*: 0.00) (Table 3). In addition, subgroup analyses based on the diagnostic criteria used in the primary studies, specifically clinical attachment loss, and pocket depth are presented in Table 3.

We utilized the GRADE methodology to assess the quality of evidence, and despite the observational nature of the studies, the evidence was deemed of high quality due to the large effect size observed [OR = 2.47, 95% CI: 2.01 to 3.03 (as shown in the GRADE table, Table 4)]. However, we downgraded the certainty of evidence due to funnel plot asymmetry, which implies that some negative results may be missing from the analysis.

**Table 4:** Results of the GRADE Assessment

**Summary of findings:**				
**Patient or population**: [health problem and/or population] **Setting**: Observational studies **Intervention**: Chronic Periodontitis **Comparison**: H. pylori				
Outcomes	Relative effect (95% CI)	№ of participants (studies)	Certainty of the evidence (GRADE)	Comments
Periodontal diseases and H. pylori infection association	OR 2.47 (2.01 to 3.03)	1481 cases 885 controls 775/2019 exposed, 759/4216 unexposed (23 observational studies)	⨁⨁◯◯ Low^a,b,c^	The evidence suggests that chronic periodontitis and H. pylori infection have a strong association.
*The risk in the intervention group (and its 95% confidence interval) is based on the assumed risk in the comparison group and the **relative effect** of the intervention (and its 95% CI). **CI**: confidence interval; **OR**: odds ratio				
**GRADE Working Group grades of evidence** **High certainty**: we are very confident that the true effect lies close to that of the estimate of the effect. **Moderate certainty**: we are moderately confident in the effect estimate: the true effect is likely to be close to the estimate of the effect, but there is a possibility that it is substantially different. **Low certainty**: our confidence in the effect estimate is limited: the true effect may be substantially different from the estimate of the effect. **Very low certainty**: we have very little confidence in the effect estimate: the true effect is likely to be substantially different from the estimate of effect.				
**Explanations**				

a. Adequate information size, with robust estimates

b. Asymmetry detected by funnel plot

c. An overall Odds Ratio of 2.47 [2.01, 3.03] supported by large effects in 19/23 studies (86.66% study population)

## Discussion

The main purpose of the present meta-analysis was to determine the association between the presence of *H. pylori* infection and the occurrence of the PDs. The number of studies examining the association between these two variables has doubled from 2019 to 2022, indicating that researchers are interested in this controversial association. In a previous meta-analysis, Wei et al. (2019) reviewed 11 related studies and reported that *H. pylori* increased the odd ratio of CPD by 3.42 times [7]. This result was in line with the ones of the meta-analysis in which 23 eligible studies were reviewed and analyzed. After combining the results of these studies, it was found the presence of *H. pylori* infection increased the risk of the PDs by 2.47 times. CPD leads to tooth loss by gingival inflammation, alveolar bone resorption, alveolar bone height reduction, and periodontal pocket formation. Therefore, identifying the factors affecting this disease can be useful in developing appropriate and effective care strategies. The results of a recent study on clinical trials in China showed there was little to moderate evidence of a reduction in *H. pylori* infection by periodontal therapy [26]. Given that both PDs and *H. pylori* have common risk factors such as poor socioeconomic status, poor health, smoking, alcohol consumption and uncontrolled diabetes, the presence of one of these diseases may exacerbate the other [27, 28]. Therefore, treating and eradicating one of them may reduce the other one. On the other hand, the results of previous studies showed in the presence of the PDs in people of the community, the probability of a positive serum test for *H. pylori* infection was high. This was the main challenge for primary and secondary studies, especially the present meta-analysis. Most early studies randomly took samples for the diagnosis of *H. pylori* infection from various parts of the mouth, such as coronal tooth sites, intra-oral mucosa, and unspecified periodontal pockets [5, 29, 30]. On the other hand, some other studies went deeper and below the gums to take samples for the diagnosis of *H. pylori* infection and their results showed sub-gingival plaques could act as a main reservoir for *H. pylori* infection [31, 32]. However, the results of the present meta-analysis confirmed this fact and showed there was a positive and significant association between the presence of *H. pylori* infection and the PDs. The reason for this association from the view point of public and clinical health can be attributed to the transmission of *H. pylori* because one of the most important ways of its transmission is the oral-fecal way [28, 33, 34]. For this reason, the oral environment, especially the gingival sulcus and dental plaque, can act as an extra-gastric reservoir for the bacteria [34–36]. According to the results of a meta-analysis conducted in the past, the simultaneous prevalence of *H. pylori* infection in the stomach and dental plaque was 49.7% [36].

Other reasons for this association include claims and assumptions expressed in some studies which suggest *H. pylori* routinely lives in dental plaque and under the gums or gingival sulcus and may cause gastrointestinal illness at any time. This confirms the presence of bacteria in the gums causes the PDs [37–39]. Of course, this hypothesis needs further studies.

The present meta-analysis examined the association between the presence of *H. pylori* infection and the PDs by considering all studies in the field, which included cross-sectional analytical and case-control studies. The difference between the present meta-analysis and those performed in the past was that in this meta-analysis the number of the studied populations was more than other previous secondary studies. The high number of preliminary studies allowed the researchers to report subgroup analyzes with accurate findings and narrow confidence intervals. On the other hand, the reduced heterogeneity in the subgroup analyzes was another reason for the accuracy and consistency of the results in this meta-analysis. Subgroup analyzes showed hospital-based studies overestimated the association between *H. pylori* infection and the PDs compared to population-based articles. This indicates hospital-based studies have been exposed to information bias and selection, which has led to over-estimate of the association. Meanwhile, the confidence interval obtained after combining hospital-based studies was wider than the one obtained from combining population-based studies, which also showed random errors were not controlled in hospital-based studies.

The results of the present meta-analysis showed in contrast to the general population, people with CPD were more prone to *H. pylori* infection. Periodontitis is an inflammatory process affecting the tissues around the tooth, such as connective tissues and bones. Accumulation of bacterial plaque on the surface of the tooth causes inflammation of the gums and if left untreated and progresses, it becomes chronic periodontitis characterized by the loss of tissues attached to the tooth. Finally, these conditions provide a favorable environment for the growth of *H. pylori*. Periodontitis is closely related to anaerobic bacteria such as Bacteroides forsythus, Porphyromonas gingivalis, *H. pylori* and Actinobacillus actinomycetemcomitans [40–44]. Oral bacteria alone cannot cause inflammation, and changes in the host’s immune response contribute to gingivitis and periodontitis [45]. Also, in people with chronic gastritis, the association between *H. pylori* infection and the periodontal disease was more significant. In the American population, the association between *H. pylori* infection and the PDs was greater and more significant than in other populations, such as Asian and European ones, but the remarkable point was the calculated confidence interval for the American population. This confidence interval was wide while the confidence interval obtained from the effect size of the association between *H. pylori* infection and the PDs was narrower. The calculated association was more accurate in Asian populations than in American and European ones according to the calculated confidence interval. The main reason for this can be attributed to the large number of studies conducted in Asian populations. Results of subgroup analysis in this meta-analysis showed that were used of various methods to detect PDs in the selected primary studies. Between these methods, the probing test and clinical attachment loss were used more than other methods. The heterogeneity rate in this subgroup analysis decreased considerable, that confirm the use of various methods for detecting PDs are main source of heterogeneity in overall pooled estimate.

Our research findings indicate that the risk of PD varies depending on the diagnostic criteria employed. Studies utilizing clinical examination as the diagnostic criterion showed higher odds of periodontal disease in individuals with H. pylori infection at 4.90, which was higher than other diagnostic criteria. Similarly, studies that did not provide a clear definition of periodontal disease also demonstrated a higher odds ratio. Therefore, in cases where a precise criterion for defining PD is lacking, there is a possibility of overestimating the association. It is important to note that the use of different diagnostic criteria can lead to varying risk estimates for periodontal disease, which should be taken into account during data analysis. Furthermore, the use of less reliable diagnostic criteria due to the lack of a precise definition of periodontal disease in some studies may result in inaccurate outcomes.

The certainty of evidence was rated as low according to the GRADE system, primarily due to the absence of a standardized diagnostic criteria for chronic periodontitis, which hindered the ability to improve the level of evidence. Furthermore, our subgroup analysis did not reveal any significant dose-response correlation with either clinical attachment loss or depth of pocket, partially due to the limited number of studies that provided such data, and the fact that neither of the criteria alone is adequate to establish a clinical diagnosis of chronic periodontitis [46].

One of the main limitations of this study was the lack of evidence of a causal association between *H. pylori* infection and the PDs. In this meta-analysis, cross-sectional analytical and case studies were reviewed and analyzed. To investigate the causal relationships in such non-interventional associations, the most important and best type of studies are cohorts not included in this meta-analysis because no cohort study with a similar subject to this meta-analysis was found. Also, the included and selected primary studies analyzed the presence of H. pylori, both in the bacterial plaque and in the gastric mucosa, in different territories and different assessment methods were used; finally, some studies did not report data. Also, these studies for assessment PDs used different methods or not reported these methods obviously. So, authors decided to done subgroup analysis based on various definition of these diseases. Results showed that rate of heterogeneity decreased by subgroup analysis. Therefore, the different definition of desired diseases in this meta-analysis has main factor to increase heterogeneity rate.

## Conclusion

Based on the results of this meta-analysis, the association between *H. pylori* infection and the PDs is evident. This association is especially high in Asian and American societies, but to more closely examine the association and specially to determine the temporal and causal association between the two diseases, carefully designing a cohort study with a large sample size is necessary.

## Supplementary Information

**Additional file 1.**

## Data Availability

The datasets used and analyzed during the current study are available from the corresponding author on reasonable request.

## Abbreviations

PDs: Periodontal Diseases
H. pylori: Helicobacter Pylori
CDP: Chronic Periodontal Diseases
PRISMA: The Preferred Reporting Items for Systematic Reviews and Meta-Analyzes
OR: Odds Ratio
CI: Confidence Interval

## References

[1] Anand PS, Nandakumar K, Shenoy K (2006). Are dental plaque, poor oral hygiene, and periodontal disease associated with Helicobacter pylori infection?. J Periodontol.

[2] Fang M (2022). Distribution characteristics of the sabA, hofC, homA, homB and frpB-4 genes of Helicobacter pylori in different regions of China. PLoS ONE.

[3] Graham DY, Moss SF (2022). Antimicrobial susceptibility testing for Helicobacter pylori is now widely available: when, how, why. Official J Am Coll Gastroenterology| ACG.

[4] Graham DY, Hernaez R, Rokkas T (2022). Cross-roads for meta-analysis and network meta-analysis of H. pylori therapy. Gut.

[5] Adler I (2014). Helicobacter pylori and oral pathology: relationship with the gastric infection. World J Gastroenterology: WJG.

[6] Berroteran A (2002). Detection of Helicobacter pylori DNA in the oral cavity and gastroduodenal system of a venezuelan population. J Med Microbiol.

[7] Wei X (2019). The association between chronic periodontitis and oral Helicobacter pylori: a meta-analysis. PLoS ONE.

[8] Butt A (2001). Randomized clinical trial of Helicobacter pylori from dental plaque. J Br Surg.

[9] Sung CE (2022). Periodontitis, Helicobacter pylori infection, and gastrointestinal tract cancer mortality. J Clin Periodontol.

[10] Sekar R, Murali P, Junaid M. Quantification of Helicobacter pylori and its oncoproteins in the oral cavity: A cross-sectional study. Oral Dis. 2023;29(4):1868–74. 10.1111/odi.14141. 10.1111/odi.14141 35092112

[11] Alkhaldi NK, Alghamdi WK, Alharbi MH, Almutairi AS, Alghamdi FT. The Association Between Oral Helicobacter pylori and Gastric Complications: A Comprehensive Review. Cureus. 2022;14(5):e24703. 10.7759/cureus.24703. 10.7759/cureus.24703 PMC9162906 35663643

[12] Mehdipour A (2022). Comparing the prevalence of Helicobacter pylori and virulence factors cagA, vacA, and dupA in supra-gingival dental plaques of children with and without dental caries: a case–control study. BMC Oral Health.

[13] Beydoun MA (2021). Helicobacter pylori, periodontal pathogens, and their interactive association with incident all-cause and Alzheimer’s disease dementia in a large national survey. Mol Psychiatry.

[14] López-Valverde N, Macedo de Sousa B, López-Valverde A, Suárez A, Rodríguez C, Aragoneses JM. Possible Association of Periodontal Diseases With Helicobacter pylori Gastric Infection: A Systematic Review and Meta-Analysis. Front Med (Lausanne). 2022;9:822194. 10.3389/fmed.2022.822194. 10.3389/fmed.2022.822194 PMC9063465 35514745

[15] Tsimpiris A (2022). Periodontitis and Helicobacter pylori infection: eradication and periodontal therapy combination. Eur J Dentistry.

[16] Salehi MR (2013). A comparison in prevalence of Helicobacter pylori in the gingival crevicular fluid from subjects with periodontitis and healthy individuals using polymerase chain reaction. J Dent Res Dent Clin Dent Prospects.

[17] Anand PS, Kamath KP, Anil S (2014). Role of dental plaque, saliva and periodontal disease in Helicobacter pylori infection. World J gastroenterology: WJG.

[18] Silva DG (2010). Presence of Helicobacter pylori in supragingival dental plaque of individuals with periodontal disease and upper gastric diseases. Arch Oral Biol.

[19] Dye BA, Kruszon-Moran D, McQuillan G (2002). The relationship between periodontal disease attributes and Helicobacter pylori infection among adults in the United States. Am J Public Health.

[20] Ozturk A (2021). Periodontal treatment is associated with improvement in gastric Helicobacter pylori eradication: an updated meta-analysis of clinical trials. Int Dent J.

[21] Page MJ (2021). The PRISMA 2020 statement: an updated guideline for reporting systematic reviews. Int J Surg.

[22] Wells G, Shea B, O’Connell D, Peterson J, Welch V, Losos M. The Newcastle-Otawa Quality Assessment Scale (NOS) for assessing the quality of nonrandomised studies in meta-analyses. 2009. [acessado em 28 jul. 2018]. Disponível em: http://www.ohri.ca/programs/clinical_epidemiology/oxford.asp.

[23] Huedo-Medina TB (2006). Assessing heterogeneity in meta-analysis: Q statistic or I² index?. Psychol Methods.

[24] Higgins JP (2008). Commentary: heterogeneity in meta-analysis should be expected and appropriately quantified. Int J Epidemiol.

[25] Gdt G. GRADEpro guideline development tool [software]. Hamilton: McMaster University; 2015.

[26] Nisha KJ, Nandakumar K, Shenoy KT, Janam P. Periodontal disease and Helicobacter pylori infection: a community-based study using serology and rapid urease test. J Investig Clin Dent. 2016;7(1):37–45. 10.1111/jicd.12122. 10.1111/jicd.12122 25175565

[27] L-X., G., *The research of relationship between periodontitis and helicobacter pylori*. J coal industry, 2011. 14(4):625–7.

[28] Zheng P, Zhou W (2015). Relation between periodontitis and helicobacter pylori infection. Int J Clin Exp Med.

[29] Miao WJ, Z.L.Z.G.C (2015). The clinical study on the effects of chronic periodontitis and periodontal initial therapy for the infection and eradication of Helicobacter pylori. Chin J Gerontol.

[30] Jing LJ (2015). Association between Helicobacter pylori infection and risk of Periodontal Diseases in Han non-smoking chinese. J dent research.

[31] Umeda M, Kobayashi H, Takeuchi Y, Hayashi J, Morotome-Hayashi Y, Yano K, Aoki A, Ohkusa T, Ishikawa I. High prevalence of Helicobacter pylori detected by PCR in the oral cavities of periodontitis patients. J Periodontol. 2003;74(1):129–34. 10.1902/jop.2003.74.1.129. 10.1902/jop.2003.74.1.129 12593608

[32] Souto R, Colombo APV (2008). Detection of Helicobacter pylori by polymerase chain reaction in the subgingival biofilm and saliva of non-dyspeptic periodontal patients. J Periodontol.

[33] Al Asqah M (2009). Is the presence of Helicobacter pylori in the dental plaque of patients with chronic periodontitis a risk factor for gastric infection?. Can J Gastroenterol.

[34] Medina ML et al. *Molecular detection of Helicobacter pylori in oral samples from patients suffering digestive pathologies* 2010. 19680173

[35] Sujatha S, Jalihal UM, Sharma S (2015). Association between periodontal disease and oral and gastric Helicobacter pylori infection. Indian J Gastroenterol.

[36] Yang J (2016). Association between Helicobacter pylori infection and risk of periodontal diseases in Han Chinese: a case-control study. Med Sci monitor: Int Med J experimental Clin Res.

[37] Tahbaz SV (2017). Occurrence of Helicobacter pylori and its major virulence genotypes in dental plaque samples of patients with chronic periodontitis in Iran Gastroenterology and hepatology from bed to bench.

[38] Riggio MP, Lennon A, Wray D (2000). Detection of Helicobacter pylori DNA in recurrent aphthous stomatitis tissue by PCR. J oral Pathol Med.

[39] W., L., *The significance of detection Helicobacter pylori in the tissue of chronic periodontitis utilizing PCR technique*. J Henan Medical College for Staff and Workers, 2001. 13(3):0248–02.

[40] Al-Refai A-NM (2002). Incidence of helicobacter pylori in dental plaque of saudi gastritis patients. J Fam Commun Med.

[41] Ying JH (2002). Effect of oral environmental changes on Helicobacter pylori in patients with periodontitis. Med Zhejiang.

[42] Jing GS, Z.X.L.Y.L.Y.W (2011). Correlativity of the chronic periodontitis with helicobacter pylori infection. J Clin Stomatol.

[43] Tsimpiris A (2021). Periodontitis and Helicobacter pylori infection: eradication and periodontal therapy combination. Eur J dentistry.

[44] Almashhadany DA, Zefenkey ZF, Zaki AM (2022). Dental risk factors associated with oral Helicobacter pylori infection: a cross-sectional study based on saliva antigen test. J Infect Developing Ctries.

[45] Ren Q, Yan X, Zhou Y, Li WX. Periodontal therapy as adjunctive treatment for gastric Helicobacter pylori infection. Cochrane Database Syst Rev. 2016;2(2):CD009477. 10.1002/14651858.CD009477.pub2. 10.1002/14651858.CD009477.pub2 PMC8255095 26852297

[46] Genco RJ, Borgnakke WS (2013). Risk factors for periodontal disease. Periodontol 2000.

