# Supplementary material for: The association between periodontal diseases and helicobacter pylori: an updated meta-analysis of observational studies
Source: BMC Oral Health. 2023 Jul 26;23:523. doi: 10.1186/s12903-023-03232-3 (PMC10369707; doi:10.1186/s12903-023-03232-3)
Supplement: Supplementary file 1 — Additional file 1. [file 12903_2023_3232_MOESM1_ESM.docx]

Table S1: the search strategy syntax

| PubMed  ((“Helicobacter pylori” OR “Helicobacter pylori infection” OR “Helicobacter nemestrinae” OR “Campylobacter pylori” OR “Campylobacter pylori subsp pylori” OR “Campylobacter pyloridis”) AND (“Periodontal Disease*” OR (Disease* AND Periodontal) OR “Periodontal Disease*” OR “Parodontosis” OR “Parodontoses” OR “Pyorrhea Alveolaris”))  Scopus  (TITLE-ABS-KEY(“Helicobacter pylori”) OR TITLE-ABS-KEY(“Helicobacter pylori infection”) OR TITLE-ABS-KEY(“Helicobacter nemestrinae”) OR TITLE-ABS-KEY(“Campylobacter pylori”) OR TITLE-ABS-KEY(“Campylobacter pylori subsp pylori”) OR TITLE-ABS-KEY(“Campylobacter pyloridis”)) AND (TITLE-ABS-KEY (“Periodontal Disease*” OR (TITLE-ABS-KEY (Disease*) AND (TITLE-ABS-KEY (Periodontal)) OR TITLE-ABS-KEY (“Periodontal Disease*”) OR TITLE-ABS-KEY (“Parodontosis”) OR TITLE-ABS-KEY (“Parodontoses”) OR TITLE-ABS-KEY (“Pyorrhea Alveolaris”))  Web of Science  (TS=(“Helicobacter pylori”) OR TS=(“Helicobacter pylori infection”) OR TS=(“Helicobacter nemestrinae”) OR TS=(“Campylobacter pylori”) OR TS=(“Campylobacter pylori subsp pylori”) OR TS=(“Campylobacter pyloridis”)) AND (TS=(“Periodontal Disease*”) OR TS= (Disease* AND Periodontal) OR TS= (“Periodontal Disease*”) OR TS= (“Parodontosis”) OR TS= (“Parodontoses”) OR TS= (“Pyorrhea Alveolaris”)) |
| --- |

| Study | Selection | | | | Comparability | Exposure | | | Study score  /9 |
| --- | --- | --- | --- | --- | --- | --- | --- | --- | --- |
|  | Case definition | Representative | Selection of controls | Definition of controls | Comparability of cases and controls on the basis of the design or analysis | Ascertainment of exposure | Same method of ascertainment for cases and controls | Non-response rate |  |
| LX Gong, et al. | * | * | * | * | * | ** | - | * | 8 |
| Zheng P, et al. | * | * | * | * | * | ** | - | - | 7 |
| MY Wang, et al. | * | * | * | * | * | ** | - | - | 7 |
| Jing Li, et al. | * | * | * | * | * | ** | - | * | 8 |
| Anand PS, et al. | * | * | * | * | * | * | * | - | 7 |
| Al Asqah M, et al. | * | * | * | * | * | * | * | * | 8 |
| Medina ML, et al. | * | * | * | * | * | * | * | * | 8 |
| Salehi MR, et al. | * | * | * | - | * | * | - | * | 6 |
| Yang J, et al. | * | * | * | * | * | ** | - | - | 7 |
| Tahbaz SV, et al. | * | * | * | * | * | ** | - | - | 7 |
| LP Wang, et al. | * | * | * | * | * | * | * | - | 7 |
| Al-Refai AN, et al. | * | * | * | * | * | * | * | * | 8 |
| YH Jiang, et al. | * | * | * | * | - | * | * | - | 6 |
| Jing Gao, et al. | * | * | * | * | - | ** | * | - | 7 |
| A Tsimpiris, et al. | * | * | * | * | - | ** | * | - | 7 |

Table S2: Methodological Quality scores included case-control studies using Newcastle-Ottawa scale

Table S3: Methodological Quality scores included cross-sectional studies using Newcastle-Ottawa scale

| Study | Selection | | | Comparability | Outcome | | Study score  /9 |
| --- | --- | --- | --- | --- | --- | --- | --- |
|  | Representativeness of the sample | Non-respondents | Ascertainment of the exposure | The subjects in different outcome groups are comparable, based on the study design or analysis. Confounding factors are controlled. | Assessment of the outcome | Statistical test |  |
| Nisha KJ, et al. | * | * | * | ** | ** | - | 7 |
| Dye BA, et al. | * | * | * | ** | ** | * | 8 |
| Umeda M, et al. | * | * | * | ** | ** | - | 7 |
| Souto R, et al. | * | * | * | ** | ** | * | 8 |
| Silva DG, et al. | * | * | * | * | * | * | 6 |
| Sujatha S, et al. | * | * | * | ** | * | * | 7 |
| Riggio MP, et al. | * | * | * | ** | * | * | 7 |
| Almashhadany DA, et al | * | * | * | ** | ** | - | 7 |
